# Self-Reported Data and Physician-Reported Data in Patients With Eosinophilic Granulomatosis With Polyangiitis: Comparative Analysis

**DOI:** 10.2196/27273

**Published:** 2022-05-25

**Authors:** Irena Doubelt, Jason M Springer, Tanaz A Kermani, Antoine G Sreih, Cristina Burroughs, David Cuthbertson, Simon Carette, Nader A Khalidi, Curry L Koening, Carol Langford, Carol A McAlear, Larry W Moreland, Paul A Monach, Dianne G Shaw, Philip Seo, Ulrich Specks, Kenneth J Warrington, Kalen Young, Peter A Merkel, Christian Pagnoux

**Affiliations:** 1 Vasculitis Clinic Mount Sinai Hospital Toronto, ON Canada; 2 Division of Rheumatology University of Toronto Toronto, ON Canada; 3 Division of Rheumatology and Immunology Medical Center Vanderbilt University Nashville, TN United States; 4 Division of Rheumatology University of California, Los Angeles Los Angeles, CA United States; 5 Division of Rheumatology University of Pennsylvania Philadelphia, PA United States; 6 Health Informatics Institute University of South Florida Tampa, FL United States; 7 Division of Rheumatology McMaster University and St. Joseph’s Healthcare Hamilton, ON Canada; 8 Division of Rheumatology University of Utah Hospital Salt Lake City, UT United States; 9 Division of Rheumatology Cleveland Clinic Cleveland, OH United States; 10 Division of Rheumatology University of Pittsburgh Pittsburgh, PA United States; 11 Division of Rheumatology Veterans Affairs Boston Healthcare System Boston, MA United States; 12 Vasculitis Foundation Kansas City, MO United States; 13 Division of Rheumatology Johns Hopkins University Baltimore, MD United States; 14 Division of Pulmonary and Critical Care Medicine College of Medicine and Science Mayo Clinic Rochester, MN United States; 15 Division of Rheumatology College of Medicine and Science Mayo Clinic Rochester, MN United States

**Keywords:** eosinophilic granulomatosis with polyangiitis, patient-reported outcomes measures, clinical outcomes, granulomatosis, patient outcomes, digital health, health network, health databases, research network

## Abstract

**Background:**

Patient-based registries can help advance research on rare diseases such as eosinophilic granulomatosis with polyangiitis (EGPA), a complex multiorgan form of antineutrophil cytoplasmic antibody (ANCA)–associated vasculitis.

**Objective:**

The aim of this study is to compare patient-reported and physician-reported data on manifestations, treatments, and outcomes for patients with EGPA.

**Methods:**

We completed a comparative analysis of patients ≥18 years with EGPA in Canada and the United States from the following 2 cohorts: (1) The Vasculitis Patient-Powered Research Network (VPPRN), a self-enrolled secure portal with patient-entered data updated quarterly (2014-2019) and (2) the Vasculitis Clinical Research Consortium (VCRC) observational studies, a physician-entered database (2003-2019) of patients who fulfilled the 1990 American College of Rheumatology classification criteria for EGPA. The studied parameters included demographic characteristics, clinical manifestations, ANCA status, treatments, and relapses.

**Results:**

Data from 195 patients with a validated diagnosis of EGPA in the VPPRN and 354 patients enrolled in the VCRC were analyzed. Compared to the VCRC cohort, the patients in the VPPRN cohort were more likely to be female (135/195, 69.2% compared to 209/354, 59%; *P*=.02) and younger at diagnosis (47.3 compared to 50.0 years; *P*=.03); both cohorts reported similar frequencies of asthma (177/184, 96.2% in the VPPRN cohort compared to 329/354, 92.9% in the VCRC cohort; *P*=.13) and cardiac manifestations (44/153, 28.8% compared to 75/354, 21.2%; *P*=.06), but the VPPRN cohort reported less frequent lung manifestations other than asthma and more frequent disease manifestations in all other organ systems. The ANCA positivity was 48.9% (64/131) in the VPPRN patients compared to 38.9% (123/316; *P*=.05) in the VCRC cohort. Relapsing disease after study enrollment was reported in 32.3% (63/195) of patients in the VPPRN compared to 35.7% (99/277) of patients in the VCRC. Most therapies (GC, cyclophosphamide, mepolizumab) were used at similar frequencies in both groups, except for rituximab with VPPRN patients reporting more use than the VCRC cohort (47/195, 24.1% compared to 29/277, 10.5%; *P*<.001).

**Conclusions:**

Overall, patients and physicians report manifestations of EGPA at similar frequencies. However, observed differences between patient and physician reports imply the potential occurrence of selection biases. These results support the use of patient-reported data in EGPA but also the need for careful consideration of disease-specific definitions for the study of EGPA and how patient- and physician-reported data are collected.

**Trial Registration:**

ClinicalTrials.gov NCT00315380, https://clinicaltrials.gov/ct2/show/NCT00315380; ClinicalTrials.gov NCT01241305, https://clinicaltrials.gov/ct2/show/NCT01241305

## Introduction

Vasculitides are rare, heterogeneous, multisystem diseases causing inflammation of blood vessels [1]. Physicians determine disease activity, damage, and prognosis in vasculitis using various clinical, laboratory, or radiological parameters or tools, such as the Birmingham Vasculitis Activity Score [2] or the Vasculitis Damage Index [3]. Many of these parameters may at times differ from the patient’s subjective disease experiences. There is growing interest in increasing patient engagement in health care research to improve the alignment of patients’ and physicians’ perspectives in the diagnosis, management, and assessment of outcomes and burden of disease; more and more action has been taken to achieve this. [4].

The Vasculitis Patient-Powered Research Network (VPPRN), an international, internet-based, prospective longitudinal registry of patient- or caregiver-reported information, was launched in 2014 to support people with any form of vasculitis by involving them in clinical research [4-6]. With over 3000 members enrolled to date, mostly from North America, it maintains a secure web-based registry where patients provide clinical data about themselves and their condition regarding demographic characteristics, diagnosis, disease extent, medications, and outcomes [4].

The Vasculitis Clinical Research Consortium (VCRC), established in 2003, has been collecting longitudinal data in patients with various vasculitides across 8 US and 2 Canadian sites. VCRC site investigators collected similar clinical information as the VPPRN.

This study aimed to compare patient self-reported and physician-reported clinical manifestations, treatments, and outcomes in patients with eosinophilic granulomatosis with polyangiitis (EGPA), one of the antineutrophil cytoplasmic antibody (ANCA)–associated vasculitides. EGPA is a complex multisystem disorder that can involve any combination of many manifestations, especially including asthma, rhinosinusitis, eosinophilia, and vasculitis in various organs. Research on EGPA has not been as extensively conducted compared to several other forms of vasculitis. A better understanding of the utility of patient-based research registries could help advance research on this rare disease.

## Methods

### Vasculitis Patient-Powered Research Network: Patient-Driven Cohort

The VPPRN provides a secure portal through which patients self-enroll and self-report information longitudinally using the internet-based platform, as previously described [6]. Data from patients in the VPPRN (2014-2019) who self-identified as being age 18 years or older, living in Canada or the United States, and having EGPA were used for this analysis. For validation of the diagnosis, patients were excluded if they indicated that the diagnosis of EGPA was not made by a doctor and/or if they reported never having used systemic glucocorticoids (GC).

Standardized questions were used to obtain data on demographic characteristics (age, sex, ethnicity), signs and symptoms of vasculitis at any time after disease onset, diagnostic tests, prescribed treatments, and outcomes from patients, with quarterly updates by email reminders. Questions related to disease manifestations were asked using lay terms, as listed in Table 1. Patients could select responses of yes, no, or I don’t know; blank responses were excluded from the data analysis.

### Vasculitis Clinical Research Consortium: Physician-Driven Cohort

Data from patients with EGPA entered into the VCRC database, as part of either the VCRC Longitudinal Study (LS; NCT00315380) [7] or the One-Time DNA (OT) Study (NCT01241305; conducted 2013-2019) [8], were used for this analysis. Patients were ages 18 years or older at enrollment and met the 1990 American College of Rheumatology classification criteria for EGPA [9]. In the VCRC-LS observational cohort, participants had in-person assessments with site investigators at either quarterly or annual visits, based on each patient’s preference and availability, with data collection of clinical and laboratory information. Patients in the VCRC-OT were assessed only at a single study visit (at diagnosis or later). All study visits involved the completion of standardized forms that collected information on patient demographic characteristics, disease characteristics, relapse(s) prior to enrollment, and, for LS only, treatments received, relapses after enrollment, and disease-related damage (from the disease itself or treatment).

Patients may be enrolled in both databases (VPPRN and VCRC); however, at present, to comply with regulations protecting health information, the databases are not linked.

### Data Elements

Demographic characteristics, main clinical manifestations of EGPA (from disease diagnosis to data extraction), ANCA status, follow-up duration, relapses (from diagnosis and/or after enrollment), and all treatments ever received (GC and other immunosuppressive drugs) were analyzed for both cohorts.

### Ethics Approval

The VCRC study protocols were approved by the local hospital research ethics board committees at all participating VCRC sites. The VPPRN data collection protocol was approved by the institutional review board at the University of South Florida (Pro00018514_CR000001). All subjects in both the VCRC and VPPRN provided consent for their participation prior to enrollment. All research was performed in accordance with the ethical principles that have their origin in the Declaration of Helsinki.

### Statistical Analyses

Descriptive statistics were computed by calculating the mean and SD for quantitative variables and count (percent) for categorical variables. Quantitative variables were compared using unequal variances *t* tests; categorical variables were compared using chi-square tests. Statistically significant differences were defined as those with *P* values ≤.05. Statistical analyses were performed using Stata (version 12; StataCorp).

## Results

### Vasculitis Patient-Powered Research Network: Patient Characteristics

At the time of data extraction, a total of 208 patients were registered in the VPPRN with a diagnosis of EGPA. A total of 13 patients were excluded as they indicated they had not been diagnosed by a doctor and/or had never been treated with systemic GC. Of the final 195 patients, 69.2% (n=135) were female; the average age at diagnosis was 47.3 years; 176 (90.3%) were White, 4 (2.1%) were Asian, and 1 (0.5%) was Black or African American. Additional cohort-specific details are presented in Table 1.

For the 195 patients, the methods by which their diagnoses of EGPA were determined comprised of the following: symptoms (n=178, 91.3%), laboratory testing (n=157, 80.5%), biopsy results (n=98, 50.3%), and imaging (n=98, 50.3%).

### Vasculitis Clinical Research Consortium: Patient Characteristics

At the time of data extraction, the VCRC cohort included 354 patients (277 LS and 77 OT). Of the 354 patients, 59% (n=209) were female; the average age at diagnosis was 50.0 years; 309 (87.3%) were White, 20 (5.6%) were Asian, and 7 (2%) were Black or African American. Additional cohort-specific details are presented in Table 1.

### Comparisons Between Patients with EGPA in the VPPRN and VCRC

Comparisons of demographic characteristics, ANCA status, clinical manifestations (from disease diagnosis to data extraction), relapses, and all treatments ever used for patients in the VPPRN and VCRC are shown in Table 1. Compared to the patients in the VCRC, patients in the VPPRN were younger at the time of diagnosis and reported similar frequencies of asthma and cardiac manifestations, less frequent lung manifestations other than asthma, and more frequent disease manifestations in all other organ systems. Relapse rates post enrollment were similar between the 2 cohorts.

**Table 1:** A comparison of the clinical characteristics of the Vasculitis Patient-Powered Research Network and Vasculitis Clinical Research Consortium eosinophilic granulomatosis with polyangiitis cohorts.

Characteristics		VPPRN^a^ cohort (N=195)	VCRC^b^ cohort (N=354)	*P* value
**Sex, n (%)**				
	Female	135 (69.2)	209 (59)	.02
	Male	60 (30.8)	145 (40.9)	.02
Age at diagnosis in years, mean (SD)		47.3 (14.3)	50.0 (14.2)	.03
Positive test for ANCA^c^, n (%)^d^		64 (48.9)	123 (38.9)	.05
**Manifestations, n (%)^e,f^**				
	Asthma	177 (96.2)	329 (92.9)	.13
	Coughed up blood or bleeding in the lungs/alveolar hemorrhage	24 (14.4)	21 (5.9)	.001
	Problems with your lungs/lung involvement	126 (72.4)	296 (83.6)	.003
	Problems with your nose or sinuses/nasal involvement	165 (92.2)	292 (82.5)	.003
	Fever	82 (55.4)	62 (17.5)	<.001
	Weight loss	95 (55.6)	106 (29.9)	<.001
	Severe joint pain or swelling/arthralgia(s)	116 (67.1)	140 (39.5)	<.001
	Rash/skin	125 (70.6)	106 (29.9)	<.001
	Inflammation of the heart lining/cardiac	44 (28.8)	75 (21.2)	.06
	Problems with your kidneys/renal disease	39 (22.4)	36 (10.2)	<.001
	Numbness, tingling, trouble moving arms, hands, legs, or feet, or other forms of nerve damage/neurological	155 (87.6)	214 (60.5)	<.001
	Inflammation in one or both eyes that required treatment/eye disease	42 (26.1)	31 (8.8)	<.001
	Loss of blood supply to intestines or perforation/mesenteric ischemia	10 (6.1)	7 (2)	.02
	Thrombosis	24 (14)	24 (6.8)	.007
**Follow-up time in years, mean (SD)**				
	From diagnosis	8.0 (6.8)	7.0 (6.2)	.08
	From enrollment	2.2 (1.1)	3.6 (3.5)	<.001
**Relapses, n (%)**				
	Total since diagnosis	N/A	175 (49.4)	N/A^g^
	After enrollment	63 (32.3)	99 (35.7)^h^	.44
Deaths, n (%)		0 (0)^i^	11 (4)^h^	N/A
**Treatments ever received, n (%)^j^**				
	Systemic glucocorticoids	195 (100)	354 (100)	.99
	Cyclophosphamide	79 (40.5)	115 (41.5)^h^	.83
	Mepolizumab	20 (10.3)	25 (9)^h^	.65
	Rituximab	47 (24.1)	29 (10.5)^h^	<.001

^a^VPPRN: Vasculitis Patient-Powered Research Network.

^b^VCRC: Vasculitis Clinical Research Consortium.

^c^ANCA: antineutrophil cytoplasmic antibody.

^d^For this category, N=131 for the VPPRN cohort and N=316 for the VCRC cohort. Percentages have been calculated accordingly.

^e^For the VPPRN cohort, the N value for each category is the number of patients who responded yes or no (the response of “I don’t know” was excluded). The N values are as follows: asthma (N=184); coughed up blood or bleeding in the lungs (N=166); problems with your lungs (N=174); problems with your nose or sinuses (N=179); fever (N=148); weight loss (N=171); severe joint pain or swelling (N=173); rash (N=177); inflammation of the heart lining (N=153); problems with your kidneys (N=174); numbness, tingling, trouble moving arms, hands, legs, or feet, or other forms of nerve damage (N=177); Inflammation in one or both eyes that required treatment (N=161); loss of blood supply to intestines or perforation (N=165); thrombosis (N=171). The percentages have been calculated accordingly.

^f^For this category, some items are presented as follows: phrasing in VPPRN database/phrasing in VCRC database.

^g^N/A: not applicable.

^h^Data were available for 277 patients in the VCRC–Longitudinal Study (VCRC-LS). These percentages have been calculated accordingly.

^i^All patients in the VPPRN logged into the portal and completed at least 1 form within 24 months prior to data extraction with none documented as lost to follow-up; captured follow-up losses and deaths in VPPRN are limited due to the inherent nature of the study design (privacy concerns associated with contacting treating physicians, family members, etc.).

^j^Additional treatments for patients in the VCRC-LS (N=277) included azathioprine (n=145, 52.3%), methotrexate (n=109, 39.4%), and mycophenolate mofetil (n=25, 9%).

## Discussion

### Principal Findings

In this study, we compared patient-reported and physician-reported outcomes in 2 cohorts of patients with EGPA; both patients and physicians reported a spectrum of outcomes and relative frequencies of manifestations, relapse rates, and medication use that are quite consistent with what is expected for this heterogeneous multisystem disease. However, some interesting differences in how this disease was reported were also observed between the 2 cohorts, raising the possibility of selection biases impacting data collection in these 2 registries.

With the exception of asthma and cardiac involvement, patients reported, for example, higher frequencies of almost all manifestations of EGPA compared to physicians.

These differences could be due to any combination of several reasons, including the following: (1) patients over-report manifestations, some of which may not be due to EGPA; (2) physicians under-report manifestations and/or do not validate patients’ reports of problems prior to evaluating them; (3) patients and physicians have a different understanding of specific manifestations; and (4) the VPPRN and VCRC involved 2 separate cohorts due to selection and inclusion biases.

Whereas the 2 cohorts did appear similar overall to what is expected for a large group of patients with EGPA, it is possible that the patients in the VPPRN had, at least initially, more severe disease or a different disease phenotype. The VPPRN cohort had a higher proportion of ANCA-positive patients, who were shown in a few previous cohort studies to present more often with surrogates of vasculitis, such as renal or cutaneous involvement [10-13]. ANCA-positive patients may also be treated with rituximab more frequently compared to the ANCA-negative EGPA population. Due to regulations protecting health information, direct linkage and comparisons of the patient-reported and physician-reported data for those “shared” participants (patients enrolled in both studies regardless of ANCA status) were not possible.

Certain subgroups may have been overrepresented due to biased sampling, as in other patient-driven registries. The mode of survey via internet-based participation for the VPPRN cohort may have enrolled more younger and female patients and more patients with ready access to the internet [14]. Such distinctions in patient- and physician-based disease features have also been observed in other rheumatic conditions [15-18]. However, previous studies using data in the VPPRN on the 2 other types of ANCA-associated vasculitis (granulomatosis with polyangiitis and microscopic polyangiitis) provided evidence for the clinical validity of the VPPRN data, thus the likely limited impact of any selection biases [6].

Misinterpretation or the use of different definitions of disease manifestations may also have contributed to some differences or inaccurate results in both cohorts. For instance, almost all patients with EGPA have asthma, whereas 70-80% of patients responded yes to “problems with [your] lungs/ lung involvement.” Asthma is considered a comorbidity or underlying manifestation of EGPA. For disease scoring and in studies on EGPA, “lung manifestations” thus usually involve other nonasthma symptoms or manifestations, separately, such as lung infiltrates, nodules, or alveolar hemorrhage. This distinction is not always applied by physicians in studies and may be even more difficult to understand by patients. The way data and information are collected in registries is crucial and clear wording is essential to deal with such aspects of the disease, especially for studies with patient-reported data. Investigators should consider all these factors when researching EGPA, both through internet-based mechanisms and traditional clinic-based approaches.

This study provides new and valuable information from both a patient and physician perspective and has several notable strengths including the sizes of the geographically diverse study cohorts, the extent of the data, overlapping leadership of the 2 research cohorts, and the long duration of follow-up for data collection. Highly experienced investigators in the field of vasculitis selected, designed, and collected the data elements using standardized forms in the VCRC and VPPRN cohorts, and patients had direct input during each stage of the process for the development of the VPPRN forms.

### Conclusions

There is a clear mandate and many benefits for health care professionals to incorporate patient perspectives into the assessment and management of vasculitis [19]. How patients’ perspectives may correspond to physicians’ assessments remains a subject of study and uncertainty. This study comparing patient-reported and physician-reported characteristics and outcomes in patients with EGPA should encourage rethinking and refinement of how patients are recruited and how their data are collected for complex diseases. These results support the use of patient-reported data in EGPA but also the need for careful consideration of these 2 types of registries, how patient- and physician-reported data are collected, as well as disease-specific definitions for the study of EGPA. Establishing a common set of disease-specific items and outcomes in EGPA and using similar definitions or wording is advised when seeking to combine data from both patients and physicians.

## Abbreviations

ANCA: antineutrophil cytoplasmic antibody
EGPA: eosinophilic granulomatosis with polyangiitis
GC: glucocorticoids
LS: longitudinal study
NCATS: National Center for Advancing Translational Science
OT: one-time DNA
VCRC: Vasculitis Clinical Research Consortium
VPPRN: Vasculitis Patient-Powered Research Network
